# A Novel Strategy for Preventing Posttransplant Large-For-Size Syndrome in Adult Liver Transplant Recipients: A Pilot Study

**DOI:** 10.3389/ti.2021.10177

**Published:** 2022-01-12

**Authors:** Xingyu Pu, Diao He, Anque Liao, Jian Yang, Tao Lv, Lunan Yan, Jiayin Yang, Hong Wu, Li Jiang

**Affiliations:** ^1^ Liver Transplantation Center, Department of Liver Surgery, West China Hospital of Sichuan University, Chengdu, China; ^2^ Laboratory of Liver Transplantation, Frontiers Science Center for Disease-Related Molecular Network, West China Hospital of Sichuan University, Chengdu, China; ^3^ Anesthesia and Operating Centre, West China Hospital of Sichuan University, Chengdu, China; ^4^ Department of General Surgery, West China TianFu Hospital, Sichuan University, Chengdu, China

**Keywords:** large-for-size syndrome, reduced-size liver transplantation, *ex vivo* right posterior sectionectomy, size mismatch, right anteroposterior vertical distance, graft-recipient weight ratio

## Abstract

There are two causes of graft compression in the large-for-size syndrome (LFSS). One is a shortage of intra-abdominal space for the liver graft, and the other is the size discrepancy between the anteroposterior dimensions of the liver graft and the lower right hemithorax of the recipient. The former could be treated using delayed fascial closure or mesh closure, but the latter may only be treated by reduction of the right liver graft to increase space. Given that split liver transplantation has strict requirements regarding donor and recipient selections, reduced-size liver transplantation, in most cases, may be the only solution. However, surgical strategies for the reduction of the right liver graft for adult liver transplantations are relatively unfamiliar. Herein, we introduce a novel strategy of HuaXi-*ex vivo* right posterior sectionectomy while preserving the right hepatic vein in the graft to prevent LFSS and propose its initial indications.

## Graphical Abstract

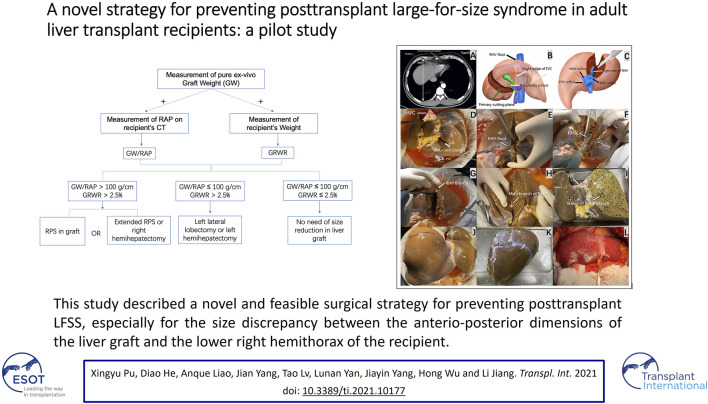

## Introduction

Large-for-size syndrome (LFSS) usually occurs in paediatric liver transplantation (LT) due to the implantation of an excessively large liver graft into a small recipient cavity, resulting in poor graft or recipient outcomes.(1, 2) However, in recent years, with the increased prevalence of obesity epidemic among the donor pool, the incidence of LFSS tends to increase in adult LTs.(3) In addition, the present organ-allocation system is mainly based on scores reflecting the severity of liver disease without any consideration of the morphological parameter mismatch between the donor and recipient.(4) Therefore, transplant surgeons can encounter graft-recipient size mismatch in adult LTs.

There are two causes of graft compression in LFSS. One is a shortage of intra-abdominal space for the graft, and the other is the size discrepancy between the anteroposterior dimensions of the graft and the lower right hemithorax of the recipient. The former could be treated using delayed fascial closure or mesh closure; however, the latter may only be treated by reduction of the right liver graft to increase space. Given that split liver transplantation (SLT) has strict requirements for donor and recipient selections,(5) reduced-size liver transplantation (RSLT), in most cases, may be the only solution. A short review of the literature(6-9) regarding the standard techniques used for graft reduction is listed in Table 1. Herein, we introduce a novel strategy of *ex vivo* right posterior sectionectomy (eRPS) while preserving the right hepatic vein (RHV) in the graft to prevent LFSS and propose its initial indications.

**TABLE 1:** A short review of the literature regarding graft reduction.

Author	Year	Recipient age (year)	Recipient gender	GRWR (%)	Reduced-size method	Surgery time (min)	Blood loss (ml)	PHS (day)	Outcome
Kim et al. (6)	2019	44	Female	3.49%	*in vivo* left lateral sectionectomy	NA	NA	45	IVC stenosis and liver and kidney dysfunction
Nagatsu et al(7)	2017	58	Female	2.74%	*in vivo* right posterior sectionectomy	554	935	21	No complication
Kim et al(8)	2015	36	Female	3.98%	*in vivo* right hemihepatectomy	386	14,000	NA	No complication
Eldeen et al(9)	2013	49	Female	NA	*ex vivo* left lateral segmentectomy	NA	NA	NA	Death due to sepsis and multiorgan failure

GRWR, graft-recipient weight ratio; IVC, inferior vena cava; NA, not available; PHS, postoperative hospital stay.

## Methods

It is dangerous for donors to undergo computed tomography (CT) examinations during organ maintenance in the intensive care unit (ICU), although CT is the most accurate method to measure the graft’s right anteroposterior (RAP) vertical distance and the largest horizontal distance. Hence, in our centre, we do not perform CT imaging on donors to ensure the safety of donors during organ maintenance in the ICU. eRPS was performed in five grafts between January 2019 and November 2020.

**FIGURE 1:**
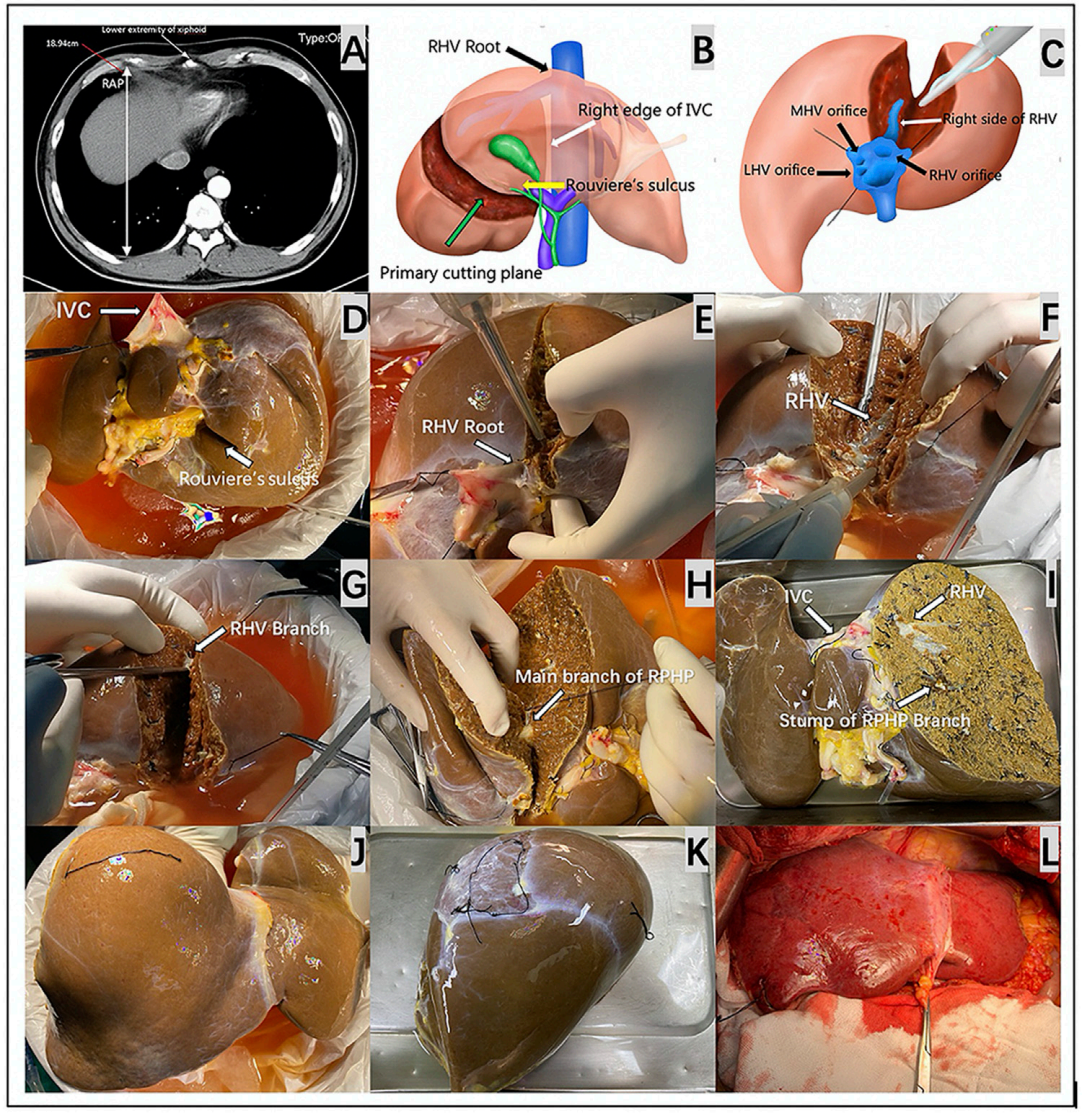
The key preoperative assessment and surgical procedures for HuaXi-eRPS. **(A)** The longest RAP vertical distance between the anterior and posterior parts of the ribs at the lower extremity of the xiphoid process is preoperatively measured on a CT scan for the recipient. **(B)** The primary cutting plane for HuaXi-eRPS is designed according to the right side of the RHV root (black arrow) entering into the suprahepatic IVC, right edge of the retrohepatic IVC (white arrow), and Rouviere’s sulcus (yellow arrow). **(C)** Parenchymal transection is designed to be started from the cranial side of the main RHV to the caudal direction, and the right side of the RHV (white arrow) is used as the surgical marker to navigate the intrahepatic transection. **(D)** The view on the visceral surface of the whole liver graft. IVC (long arrow); Rouviere’s sulcus (short arrow). **(E)** Parenchymal transection is started from the cranial side of the main RHV root (arrow) to the caudal direction. **(F)** The right side of the RHV (arrow) is used as the surgical marker to navigate the intrahepatic transection. **(G)** Dissection of the RHV branch (arrow) entering into segment VI. **(H)** Dissection of the main branch of RPHP (arrow). **(I)** The view on the visceral surface of the remnant liver graft after HuaXi-eRPS. **(J)** The view on the diaphragmatic surface of the remnant liver graft after HuaXi-eRPS. **(K)** The view on the diaphragmatic surface of the resected right posterior sector. **(L)** Implantation of the reduced-size liver graft into the recipient. HuaXi-eRPS, HuaXi-*ex vivo* right posterior sectionectomy; IVC, inferior vena cava; LHV, left hepatic vein; MHV, middle hepatic vein; RAP, right anteroposterior; RHV, right hepatic vein; RPHP, right posterior hepatic pedicle.

**FIGURE 2:**
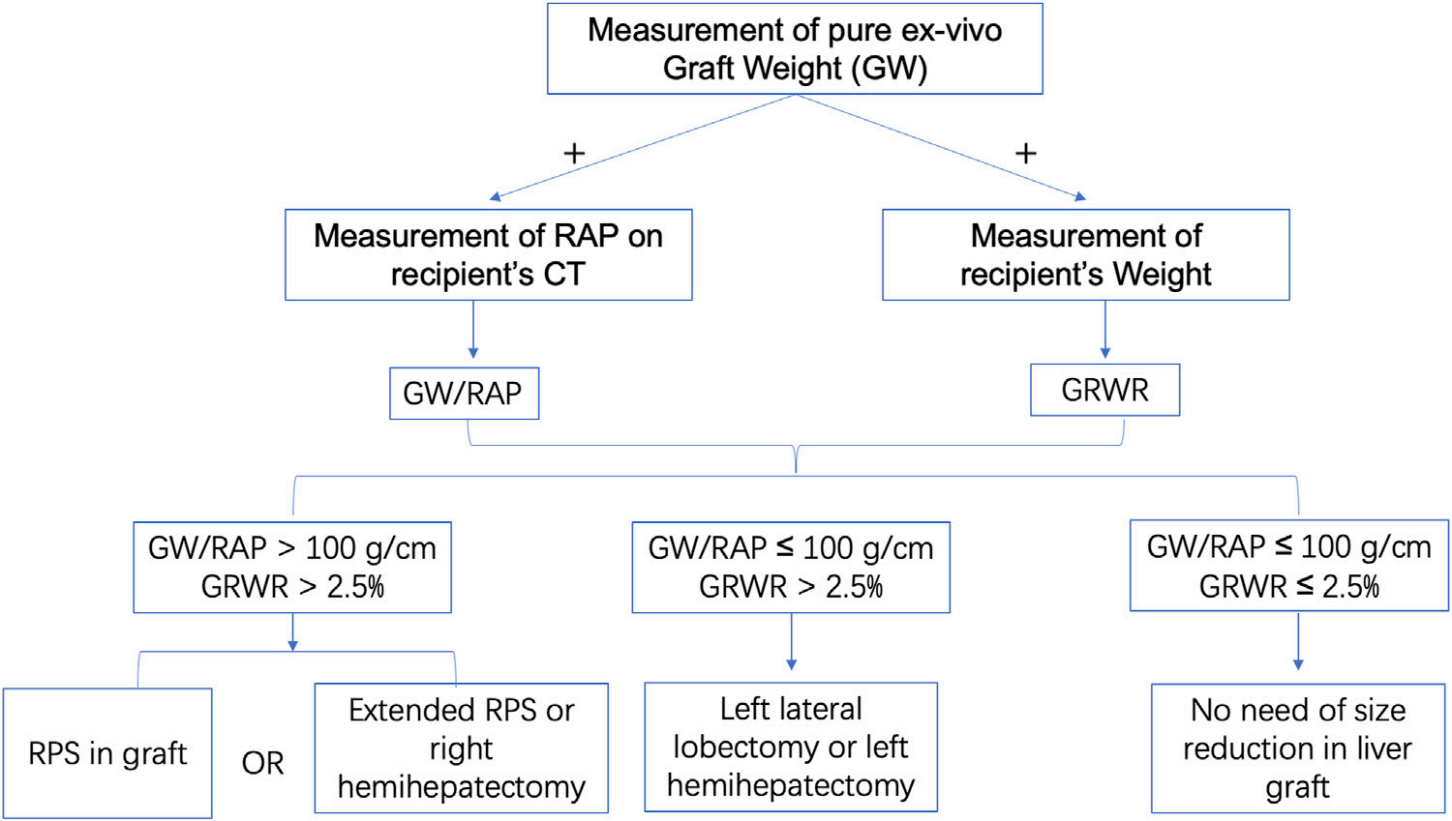
The flow chart of using GW/RAP and GRWR. First, we calculate the GW/RAP and GRWR. Subsequently, if GW/RAP > 100 g/cm and GRWR > 2.5%, RPS or extended RPS or right hemihepatectomy will be considered in graft; if GW/RAP ≤ 100 g/cm and GRWR > 2.5%, left lateral lobectomy or left hemihepatectomy will be considered in graft; if GW/RAP ≤ 100 g/cm and GRWR ≤ 2.5%, no size reduction will be considered in graft. GRWR, graft-recipient weight ratio; GW/RAP, graft weight/right anteroposterior vertical distance; RPS, right posterior sectionectomy.

Regarding the recipients, we defined the longest RAP vertical distance between the anterior and posterior parts of the ribs at the lower extremity of the xiphoid process on a CT scan (Figure 1A). Both graft-recipient weight ratio (GRWR) > 2.5% and graft weight (GW)/RAP > 100 g/cm indicated the need for reduction of the right liver graft. The estimated mean volume of the right posterior sector was approximately 27.9% of the total liver volume.(10) Based on these parameters, we can estimate the weight of the remnant graft after eRPS and if both new GRWR and GW/RAP could be reduced to normal values (≤2.5% and 100 g/cm, respectively). Therefore, it was considered acceptable to perform the eRPS. A detailed flow chart is shown in Figure 2.

All organs were donated after death, and no organs were obtained from executed prisoners. eRPS was performed on the back table. The primary cutting plane was designed according to the right side of the RHV root into the suprahepatic inferior vena cava (IVC), right edge of the retrohepatic IVC, and Rouviere’s sulcus (Figure 1B). Parenchymal transection was started from the cranial side of the main RHV to the caudal direction, which was similar to the cranial approach in laparoscopic anatomic liver resection (Figure 1C). We mainly used the right side of the RHV as the surgical marker to navigate the intrahepatic transection. The cutting point for the main branch of the right posterior hepatic pedicle (RPHP) was in Rouviere’s sulcus and was distant from the porta hepatis, which may prevent damage to the right anterior hepatic pedicle (Figures 1D–I). Cavitron ultrasonic surgical aspirator combined with a harmonic scalpel was used to dissect the liver parenchyma, and intrahepatic larger ducts of more than 3 mm were ligated or clipped. The main branch of the RPHP was clipped or transected using a linear stapler. Hemostasis was achieved using the Aquamantys System (Medtronic Advanced Energy, United States). Any potential leaks were carefully detected via repeated organ perfusion and sutured before implantation, and the bile leak test was completed at the back table by injecting indocyanine green into the graft’s bile duct. Finally, the right posterior sector and remnant grafts were weighed separately. (Figures 1J,K). All reduced grafts were implanted using the piggyback method (Figure 1L). Owing to the innovation of this technology, we named it HuaXi-eRPS (HuaXi is the acronym of our hospital name, West China Hospital of Sichuan University). This study was approved by the West China Hospital Ethics Committee and was conducted in accordance with the ethical guidelines of the Declaration of Helsinki.

## Results

In this study, HuaXi-eRPS was performed in five grafts. The five donors did not meet the criteria for split candidates utilised by UNOS(5); thus, SLTs were not considered. All data regarding the recipients and donors are summarised in Table 2. It took much time to separate the abdominal adhesions for three recipients with recurrent hepatocellular carcinoma (HCC) (Cases 1, 4, and 5) after liver resection. One recipient (Case 3) with fulminant hepatitis B had portal vein thrombosis and had undergone thrombectomy. In addition, meticulous hemostasis on the graft cutting face is a critical procedure for RSLT. Based on the reasons mentioned above, the total operation time was longer than that of non-RSLT.

**TABLE 2:** The related data of recipients and their allocated donors.

Parameters	Case 1	Case 2	Case 3	Case 4	Case 5
**Recipient profiles**					
Age, years	56	39	18	51	65
Gender	M	M	F	F	M
Height, cm	163	168	160	162	168
Weight, kg	67	53	59	53	54
BMI, kg/m^2^	25.22	18.78	23.05	20.2	19.13
Indications for liver transplantation	HCC recurrence	FHB	FHB	HCC recurrence	HCC recurrence
MELD scores	22	25	28	26	27
**Allocated DCD donor profiles**					
Age, years	43	62	56	54	58
Gender	M	M	M	M	M
Height, cm	180	178	175	175	176
Weight, kg	99	80	83	80	81
BMI, kg/m^2^	30.56	25.25	27.1	26.12	26.15
Death reason	Acute cerebral hernia	Acute cerebral hernia	Cerebral hemorrhage	Irreversible cerebral injury	Irreversible cerebral injury
**Intraoperative data**					
Procured GW, g	2060	1830	1750	1800	1850
Preoperatively measured RAP in recipients, cm	18.94	16.13	15.57	17.86	16.55
Calculated GRWR for whole graft, %	3.07	3.45	2.97	3.40	3.43
Calculated GW/RAP for whole graft, g/cm	108.8	113.5	112.4	100.8	111.8
Preoperatively estimated GRWR for the remnantt graft after eRPS, %	2.22	2.49	2.14	2.45	2.47
Preoperatively estimated GW/RAP for the remnant graft after eRPS, g/cm	78.4	81.8	81.0	72.7	80.6
Actual weight of the remanent graft after eRPS, g	1,526	1,250	1,320	1,295	1,300
Actual GRWR after *ex vivo* reduction, %	2.28	2.36	2.24	2.44	2.41
Actual GW/RAP after *ex vivo* reduction, g/cm	80.6	77.5	84.8	72.5	78.5
Duration for graft reduction, min	40	33	41	38	35
Total operation time for recipient, h	7.5	5.9	7.7	8.2	8.5
Anhepatic time for recipient, min	85	76	75	70	74
Cold ischemic time, min	359	402	300	414	383
Estimated total blood loss, ml	650	2,100	2,250	1,120	1,020
Estimated blood loss after anhepatic phase, ml	170	340	360	230	240
Amount of blood transfusion during operation, units	3	13	14	4	6
**Postoperative course**					
Delay the fascial closure after LT	No	No	No	No	No
The POD of extubation	1	1	1	1	2
ICU stay, days	5	9	4	5	5
Postoperative hospital stay, days	9	19	16	13	15
Postoperative complication grade according to Clavien-Dindo classification					
Grade I					√^π^
Grade II			√^∮^		
Grade IIIa					
Grade IIIb					
Grade IVa					
Grade IVb					
Grade V					
Follow-up, months	14.2	10.1	8.2	7.2	2.1

M, male; F, female; BMI, body mass index; DCD, donation after citizen death; eRPS, *ex vivo* right posterior sectionectomy; FHB, fulminant hepatitis B; GRWR, graft-recipient weight ratio; GW, graft weight; HCC, hepatocellular carcinoma; ICU, intensive care unit; LT, liver transplantation; POD, postoperative day; RAP, right anteroposterior; ^∮^ need of blood transfusion; ^π^ wound infection.

The 30-days mortality was zero. Postoperative complications occurred in two patients (40%); however, complications higher than those in Clavien-Dindo grade II(11) were not observed in all patients. No patient experienced biliary leakage or postoperative haemorrhage, and no infection-related complications, including liver abscess or pulmonary infection, were identified in this series. During the follow-up period (range, 2.1–14.2 months), all patients were alive with normal daily activities, and three patients with HCC did not experience tumour recurrence with a normal alpha-fetoprotein level. All five recipients did not experience posttransplant rejection and biliary complications, such as bile leakage and biliary stricture, were not observed in any of the recipients.

## Discussion

The morphology of the right upper abdominal cavity may differ among individuals. To date, four formulas have been proposed to predict the occurrence of LFSS.(2, 12-14) However, only one formula introduced an individualised morphological measurement (RAP value) on the recipient.(2) In the present case series, we selected GW/RAP combined with GRWR as new “LFSS predictors” for the following reasons. First, the GW/RAP considers the depth of the lower right hemithorax, which directly influences rib compression in the right liver. Second, both GRWR and GW/RAP do not rely on the donor’s radiological examination, which is an almost impossible task when the donor is in critical condition. Third, GRWR can predict the risk of LFSS and is also a commonly used index for evaluating the occurrence of the small-for-size syndrome (SFSS).

Compared to paediatric RSLT,(15, 16) the surgical strategies for graft reduction in adult LTs are relatively unfamiliar. In most cases, a limited resection, such as left lateral lobectomy or left hemihepatectomy, is preferred because of its convenience.^2^ However, it is very unlikely to solve some mismatch issues because compression, due to the ribs, mainly applies to the right liver. Right hemihepatectomy has been proposed as an alternative method, but the residual left liver may be insufficient for some recipients.(8) Compared to the *in vivo* method, the HuaXi-eRPS used in our series could be a unique method with the following advantages. First, the graft weight can be accurately measured on the back table to provide a precise parameter for determining the feasibility of eRPS. Second, because the *ex vivo* graft can be rotated 360-degree, it is easy and simple to perform eRPS using the cranial approach to the RHV. Although the demarcated area for the right posterior sector cannot be displayed easily after ligating the right posterior Glisson’s sheath as an *in situ* graft, the main purpose of eRPS is to overcome size mismatch. It is not necessary to perform a precise anatomic right posterior sectionectomy, as required for hepatic malignancy. Third, eRPS in the graft before implantation is beneficial to reduce the difficulty of implantation and shorten the period for the anhepatic phase. In addition, compared to the whole right lobe, which accounts for 60–75% of the total liver volume, eRPS can ensure both the integrity of outflow and adequate residual graft volume to avoid SFSS while avoiding rib compression.

The present study had some limitations. GW/RAP combined with GRWR, as a new “LFSS predictor,” is a preliminary formula whose optimal cutoff value or predictive validity still requires further confirmation by a well-designed trial with a large sample size. However, this is the first study to propose the initial indications for HuaXi-eRPS in grafts, and its initial outcomes in our five adult series are safe and encouraging, especially in decreasing the difficulty of implantation, avoiding delayed fascial closure, shortening ICU stay, and reducing posttransplant complications.

In conclusion, this study described a novel and feasible surgical strategy for preventing posttransplant LFSS, especially for the size discrepancy between the anteroposterior dimensions of the liver graft and the lower right hemithorax of the recipient.

## Capsule Sentence Summary

This study describes a novel and feasible surgical strategy for preventing posttransplant large-for-size syndrome, especially for the size discrepancy between the anteroposterior dimensions of the liver graft and the lower right hemithorax of the recipient.

## Data Availability

The original contributions presented in the study are included in the article/supplementary material, further inquiries can be directed to the corresponding author.

## Abbreviations

LFSS, large-for-size syndrome; LT, liver transplantation; SLT, split liver transplantation; RSLT, reduced-size liver transplantation; eRPS, *ex vivo* right posterior sectionectomy; RHV, right hepatic vein; RAP, right anteroposterior; GRWR, graft-recipient weight ratio; GW, graft weight; IVC, inferior vena cava; RPHP, right posterior hepatic pedicle; CUSA, cavitron ultrasonic surgical aspirator; HuaXi-eRPS, HuaXi-*ex vivo* right posterior sectionectomy; FHB, fulminant hepatitis B; SFSS, small-for-size syndrome.
